# Hypoxia-inducible factor 1α is Essential for Macrophage-mediated Erythroblast Proliferation in Acute Friend Retrovirus Infection

**DOI:** 10.1038/s41598-017-17324-y

**Published:** 2017-12-08

**Authors:** Timm Schreiber, Theresa Quinting, Ulf Dittmer, Joachim Fandrey, Kathrin Sutter

**Affiliations:** 1 University of Duisburg-Essen, Institute of Physiology, Essen, Germany; 2 University of Duisburg-Essen, Institute of Virology, Essen, Germany

## Abstract

Macrophages are the frontline of defence against foreign microorganisms, including bacteria, parasites, and viruses. During acute viral infection, macrophages must invade the inflamed tissue toward low oxygen concentrations, where genetic cellular responses depend on hypoxia-inducible factors (HIF). In the study reported here we investigated the role of HIF-1α in macrophage function during acute retroviral infection. Wild-type and myeloid cell–specific *HIF-1α* knockout mice were infected with Friend retrovirus (FV), and immune response was analysed 7 and 10 days after infection. FV infection led to increased spleen weight in wild-type and knockout mice, whereas a profound proliferation of erythroblasts was seen only in wild-type mice. The number of spleen-infiltrating macrophages was also significantly lower in knockout animals. Macrophage invasion after FV infection in wild-type mice led to elevated amounts of activated macrophage-stimulating 1 protein that resulted in massive proliferation of erythrocyte precursor cells. This proliferation was absent from knockout mice because of impaired invasion capabilities of HIF-1α–deficient macrophages. Our study elucidated a novel mechanism of FV-induced erythrocyte precursor cell proliferation.

## Introduction

Macrophages are a diverse and important component of the innate immune system, with versatile functions in host defence and immunity. They are the frontline of defence against foreign microorganisms, including bacteria, parasites, and viruses^1–3^. During infection, the inflammatory response of macrophages comprises four stages: recognition of infection by pattern-recognition receptors (PRRs), recruitment of macrophages to infected tissue, elimination of pathogens, and restoration of tissue homeostasis.

Inflamed and diseased tissues are characterised by a dramatic loss of oxygen supply, called hypoxia. Oxygen tension in inflamed tissue reaches levels as low as 0.6% to 0.9% because of impaired local blood flow and increased oxygen consumption by recruited immune cells^4,5^. Thus, macrophages must move toward low oxygen concentrations if they are to infiltrate areas of acute inflammation^6^. Macrophages adapt rapidly to hypoxic conditions by altering gene expression. Of these hypoxia-inducible genes, 89% appear to have a common mode of regulation that involves activation of the hypoxia-inducible factor (HIF)^7^.

HIF is a heterodimer composed of an oxygen-sensitive alpha subunit and a constitutive beta subunit. Although both HIF-1α and HIF-2α (also known as Epas1) dimerise with HIF-1β to drive the expression of HIF target genes, the function of HIF-3α is less obvious^8^. Under normoxic conditions, prolyl hydroxylases (PHD1-PHD3) and von Hippel-Lindau (VHL) ubiquitylation complex target HIF-1α/2α for proteasomal degradation^9,10^. Under hypoxic conditions *in vitro*, both HIF-1α and HIF-2α accumulate in primary human macrophages and in murine bone marrow–derived macrophages (BMDMs)^11,12^, a finding suggesting that HIF may regulate macrophage functions during inflammation. Several studies have addressed the function of HIF-1α in macrophages during inflammatory and antibacterial activities^13–15^. However, little is known about the role of HIF-1α during viral infections.

Friend virus (FV) infection of susceptible adult mice is a well-established model of retroviral infections^16,17^ and is also a model for the multistage development of cancer^18^. The pathogenic FV complex is composed of the replication-competent helper virus Friend murine leukaemia virus (F-MuLV) and the replication-defective spleen focus-forming virus (SFFV), which is required for pathogenicity^19^. The first stage of FV infection is characterised by a polyclonal expansion of erythroid precursor cells (EPCs), which results in profound splenomegaly. Because of its characteristic progression and the specificity of FV for the erythrocyte lineage, several genes have been found to control susceptibility to FV-induced erythroleukaemia. Some of these genes, such as *Friend virus susceptibility 1* *FV1* and *FV4*, are necessary for the ability of FV to infect cells, whereas others (*W*, *S1*, and *FV2*) affect the regulation of expansion of infected cells^18^.

In susceptible mouse strains, FV infection develops into lethal erythroleukaemia. Disease-resistant strains can control acute infection, but they cannot completely eliminate the virus, and a chronic infection develops^20,21^. The *Friend virus susceptibility 2* (*FV2*) locus encodes the *macrophage-stimulating 1 receptor* (*MST1R*, also known as *STK* or *RON*). *FV2* appears to determine whether SFFV-infected erythroblasts proliferate in response to the retroviral envelope protein gp55. Susceptible mouse strains express a truncated form of MST1R (sf-STK) that lacks almost the entire extracellular domain but retains the transmembrane and tyrosine kinase domains.

The gp55 protein interacts directly with the erythropoietin receptor (EPOR). Binding of gp55 to EPOR, which is highly expressed in EPCs, results in receptor activation and promotes EPO-independent proliferation^22^. In susceptible mice, the gp55/EPOR complex recruits the short form of MST1R (sf-STK) as a signalling partner^23^. Constitutive activation of this complex leads to the dysregulation of proliferation, survival, and differentiation of EPCs, resulting in acute erythroblastosis. MST1R signalling in various types of cancer, including leukaemia, has been studied extensively (for review, see Yao *et al*.)^24^. MST1R can form homodimers or heterodimers with several other receptor tyrosine kinases, including hepatocyte growth factor receptor (HGFR), epidermal growth factor receptor (EGFR), and insulin-like growth factor 1 receptor (IGF1R). These cross-talks have emerged as a mechanism for the regulation of MST1R-mediated tumorigenesis^24^.

Moreover, MST1R is implicated in signalling pathways that are mediated by viral oncoproteins, such as Jaagsiekte sheep retrovirus (JSRV) and Epstein-Barr virus (EBV)^25,26^. MST1R is the receptor for MST1, which is constitutively transcribed in hepatocytes^27^, and can be isolated from serum at nanomolar concentrations^28,29^. It circulates in the blood in an uncleaved proform (pro-MST1)^30,31^, and it has been shown that physiologically important MST1 is generated only at the surface of macrophages, resulting in locally restricted levels of the cytokine^31^. In addition, it has been shown that MST1 plays an important role in EPC proliferation^32^. However, the function of MST1 in the pathogenesis of FV infection is unclear.

In this study, we investigated the effects of the transcription factor *HIF-1α* on macrophages during acute retroviral infection by using mice with a HIF-1α knockout in myeloid cells. Additionally, we addressed the role of MST1 in FV infection.

## Results

### Increased spleen weights in wild-type and *HIF-1α* knockout mice during Friend virus infection

**Figure 1:**
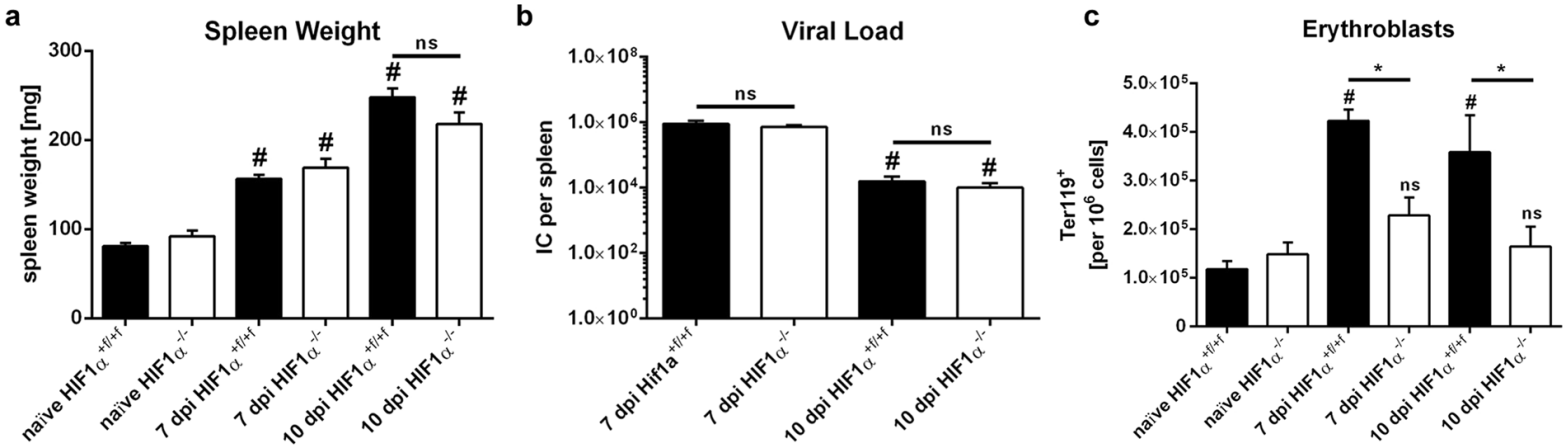
Friend virus infection leads to increased spleen weights in wild-type and hypoxia-inducible factor 1α knockout mice. Spleen weight (**a**) and viral loads (**b**) of hypoxia-inducible factor (*HIF)-1α* ^+*f/*+*f*^ and *HIF-1α* ^+*f/*+*f*^ × *LysM-Cre* mice were measured at various time points after Friend virus (FV) infection. The number of erythroblasts (Ter119^+^) was determined by flow cytometry (**c**). Data were analysed with ANOVA and Tukey’s multiple comparison test (mean + SE). n = 7–11. ^#^P = 0.05 compared to naïve mice; *P = 0.05 for wild-type (WT) mice compared with knockout (KO) mice; ns, not statistically significant.

To characterize HIF-1α function in macrophages during acute retrovirus infection, we infected *HIF-1α* ^+*f/*+*f*^ (wild-type, WT) and *HIF-1α* ^+*f/*+*f*^ × *lysosome 2 (LysM)-Cre* (knockout, KO) mice with FV for 7 or 10 days. Both WT and *HIF-1α* KO mice exhibited increases in spleen weight after infection. Spleen weight had almost doubled in WT and KO animals after 7 days and had nearly tripled after 10 days (Fig. 1a). In previous studies of FV-specific immunity, viral replication and immune responses were mainly determined in the spleens of infected mice. Therefore, we determined viral loads in the spleens of infected mice with the infectious centre assay^33^. Peak viral loads in WT and KO mice were reached at 7 days after infection (dpi) and decreased until 10 dpi (Fig. 1b). However, no differences in viral load were observed between WT and KO mice.

Next, we checked for proliferation of EPCs after FV infection by using flow cytometry analyses. The frequency of Ter119^+^ erythroblasts in WT mice increased from 11.8 ± 1.7 × 10^4^ to 42.3 ± 2.3 × 10^4^ erythroblasts per 10^6^ cells after 7 days and remained elevated after 10 days (35.5 ± 5.2 × 10^4^ erythroblasts per 10^6^ cells). In contrast, there was only a negligible, not statistically significant increase in the frequency of these cells in KO animals, from 14.9 ± 2.4 × 10^4^ to 22.9 ± 3.7 × 10^4^ (at 7 dpi) and 21.1 ± 5.3 × 10^4^ erythroblasts per 10^6^ cells (at 10 dpi; Fig. 1c). However, all infected WT and KO mice exhibited no structural alterations in splenic architecture during acute FV infection (Supplementary Fig. 1a).

Thus, FV infection resulted in increased spleen weight in WT and KO mice. However, a significant increase in the number of Ter119^+^ erythroblasts was only seen in WT mice.

### Friend virus infection leads to increased numbers of macrophages in the spleens of wild-type but not of *HIF-1α* knockout mice

**Figure 2:**
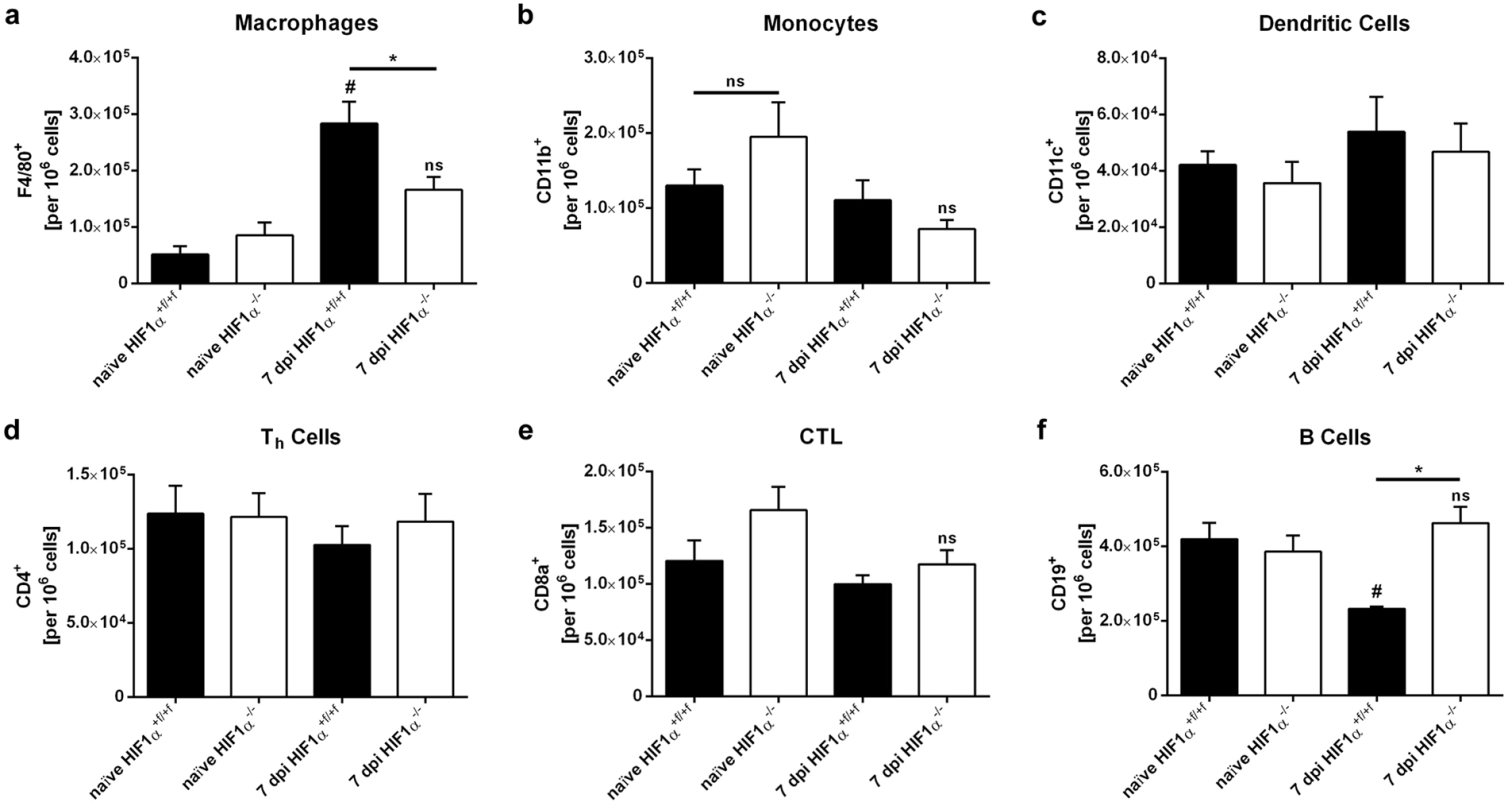
Friend virus infection leads to an increase in the number of macrophages in wild-type but not in hypoxia-inducible factor 1α knockout mice. Spleens of hypoxia-inducible factor (*HIF)-1α* ^+*f/*+*f*^ and *HIF-1α* ^+*f/*+*f*^ × *LysM-Cre* mice were isolated at 7 and 10 dpi, and subpopulations of spleen cells were analysed by flow cytometry. The following antibodies were used for cell population analysis: F4/80, macrophages (**a**); CD11b, monocytes (**b**); CD11c, dendritic cells (**c**); CD4, T helper (T_h_) cells (**d**); CD8a, cytotoxic T lymphocytes (CTLs) (**e**); CD19, B cells (**f**). Data were obtained from 2 independent experiments and were analysed with ANOVA and Tukey’s multiple comparison test (mean + SE). n = 6–16. *p-value*: 0.05, ^#^P = 0.05 compared to naïve mice; *P = 0.05 for wild-type (WT) mice compared with knockout (KO) mice; ns, not statistically significant.

To evaluate immune responses after FV infection, we performed flow cytometry analyses of spleen cells at 7 and 10 dpi. At 7 dpi there was a strong increase in the frequencies of F4/80^+^ macrophages after FV infection in WT mice (from 6.2 ± 1.6 × 10^4^ to 28.3 ± 3.9 × 10^4^ macrophages per 10^6^ cells). This increase was significantly lower in *HIF-1α* KO mice (8.5 ± 2.3 × 10^4^ to 16.6 ± 2.3 × 10^4^ macrophages per 10^6^ cells; Fig. 2a). Additionally, we observed a significant decrease in the relative numbers of CD19^+^ B cells only in WT mice after FV infection, whereas the B-cell population in KO animals remained stable (Fig. 2f). The numbers of other immune cell populations, e.g., monocytes (CD11b^+^), dendritic cells (CD11c^+^), and T cells (CD4^+^ & CD8a^+^), did not differ significantly between WT and KO animals upon infection.

A comparison of absolute numbers of total splenic cells between WT and KO mice at 7 dpi showed no statistically significant difference (Supplementary Fig. 1b). This finding may explain the increase in overall spleen weight in both animal groups (Fig. 1a). Collectively, FV infection leads to enhanced macrophage numbers in the spleen in WT mice but significantly fewer macrophages in *HIF-1α* KO mice.

### Hypoxic conditions in the spleen and HIF-1α stabilisation during Friend virus infection

**Figure 3:**
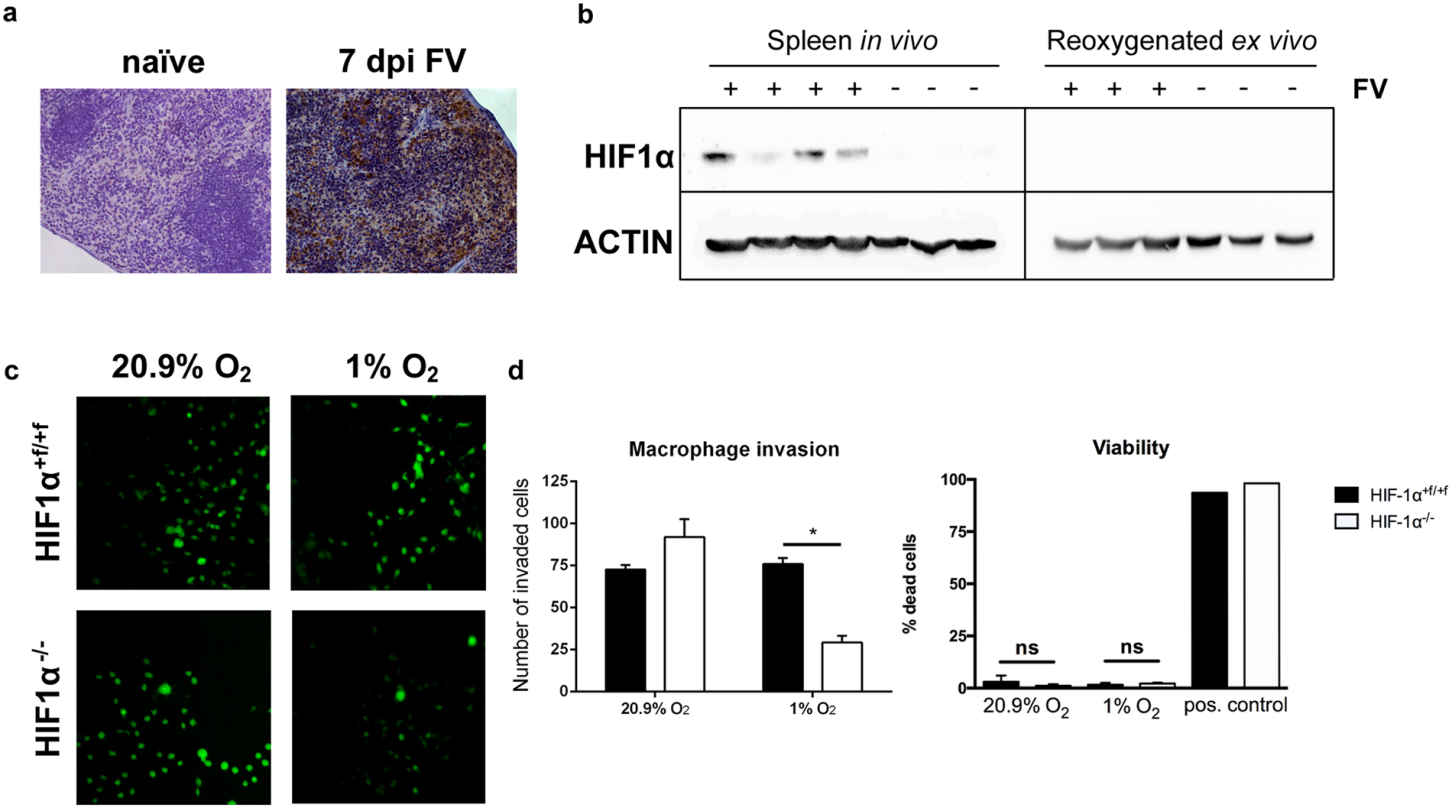
Loss of hypoxia-inducible factor 1α from macrophages impairs invasion capabilities under hypoxic conditions. Sections of spleen tissue from naïve and Friend virus (FV)-infected mice (7 days after infection [dpi]) were stained with 3,3′- diaminobenzidine (DAB) pimonidazole (brown) and then counterstained with haematoxylin (**a**, blue). Spleens of naïve and FV-infected mice (7 dpi) were removed, and proteins were directly isolated in a hypoxic workstation under hypoxic conditions (1% O_2_) so that rapid degradation of hypoxia-inducible factor (HIF) protein could be avoided. One part of the spleen was removed from the workstation and exposed to atmospheric conditions (20.9% O_2_) for 10 minutes for reoxygenation before protein isolation. Subsequently, Western blot analysis was performed with specific antibodies for HIF-1α and ACTIN (**b**). An inverted invasion assay was performed to compare 3D invasion of bone marrow–derived macrophages (BMDMs) from naïve wild-type (WT) and *HIF-1α* knockout (KO) mice under normoxic and hypoxic conditions. BMDMs were allowed to invade a Matrigel/fibronectin gel for 72 hours. Subsequently, cells were stained with calcein acetoxymethyl (AM) and visualised by confocal microscopy (**c**; original magnification, 200×). Three randomly chosen fields per well were recorded, and the number of invaded BMDMs was determined. For viability testing, dead BMDMs were stained with DAPI, and stained cells were counted manually in relation to the total number of cells in the field. Positive controls were treated with 0.1% Triton X-100. (**d**). Data were analysed with Student’s t-test (mean + SEM). n = 3–4. *P = 0.05.

Next we determined whether FV infection leads to a hypoxic environment in the spleen. To evaluate hypoxic areas in the spleen of FV-infected mice (at 7 dpi), we administered pimonidazole intraperitoneally; 60 minutes later, animals were put to death, and pimonidazole adducts produced by hypoxia were detected by immunohistochemistry^34^. Compared to naïve control mice, FV-infected mice exhibited an increase in hypoxic areas in the spleen (Fig. 3a).

Hypoxia should result in HIF-1α protein stabilisation and, thus, accumulation. To detect HIF-1α protein, we isolated the spleens of infected and naïve mice at 7 dpi by putting the mice in a hypoxia workstation under hypoxic conditions (1% O_2_) to avoid reoxygenation-dependent HIF-1α protein degradation during preparation. Western blot analyses quantified HIF-1α protein in the spleen during acute FV infection. To verify that hypoxic conditions in the spleen resulting from FV infection (Fig. 3a) were responsible for HIF-1α protein accumulation, we reoxygenated the splenic cells after preparation from the mice by exposing them to normoxic conditions (20.9% O_2_) for 10 minutes. This exposure led to rapid oxygen-dependent degradation of HIF-1α protein (Fig. 3b). Of note, no induction of *HIF-1α* mRNA expression during FV infection was detectable, a finding indicating that hypoxic stabilisation caused HIF-1α protein accumulation (Supplementary Fig. 1c). Taken together, these findings suggest that FV infection leads to a hypoxic tissue environment and to HIF-1α protein stabilisation in the spleen but neither interferes with the general ability to degrade HIF nor increases *HIF-1α* mRNA expression.

### Loss of HIF-1α from macrophages impairs invasion capabilities under hypoxic conditions

To determine how the loss of HIF-1α affects the invasion capabilities of macrophages, we used an inverted 3D invasion assay^35^. BMDMs were isolated from WT and KO mice and were allowed to invade into a Matrigel/fibronectin gel for 72 hours under normoxic (20.9% O_2_) or hypoxic (1% O_2_) conditions. After 3 days, KO and WT macrophages invaded the Matrigel comparably under normoxic conditions. Approximately similar numbers of WT BMDMs were detectable in the Matrigel under hypoxic conditions. In contrast, in *HIF-1α*–deficient BMDMs, the number of infiltrating cells decreased by more than 60% (Fig. 3c and d). To determine whether this impaired infiltration was due to a decrease in viability, we performed a 4′,6-diamidino-2-phenylindole (DAPI) exclusion test. After 72 hours, the percentage of dead cells was lower than 3% under either normoxic or hypoxic conditions. Moreover, there was no difference in the percentage of dead cells between WT and KO mice (Fig. 3d). Thus, loss of HIF-1α leads to impaired invasion of macrophages under hypoxic conditions.

### MST1 is cleaved and activated in the spleen after Friend virus infection

**Figure 4:**
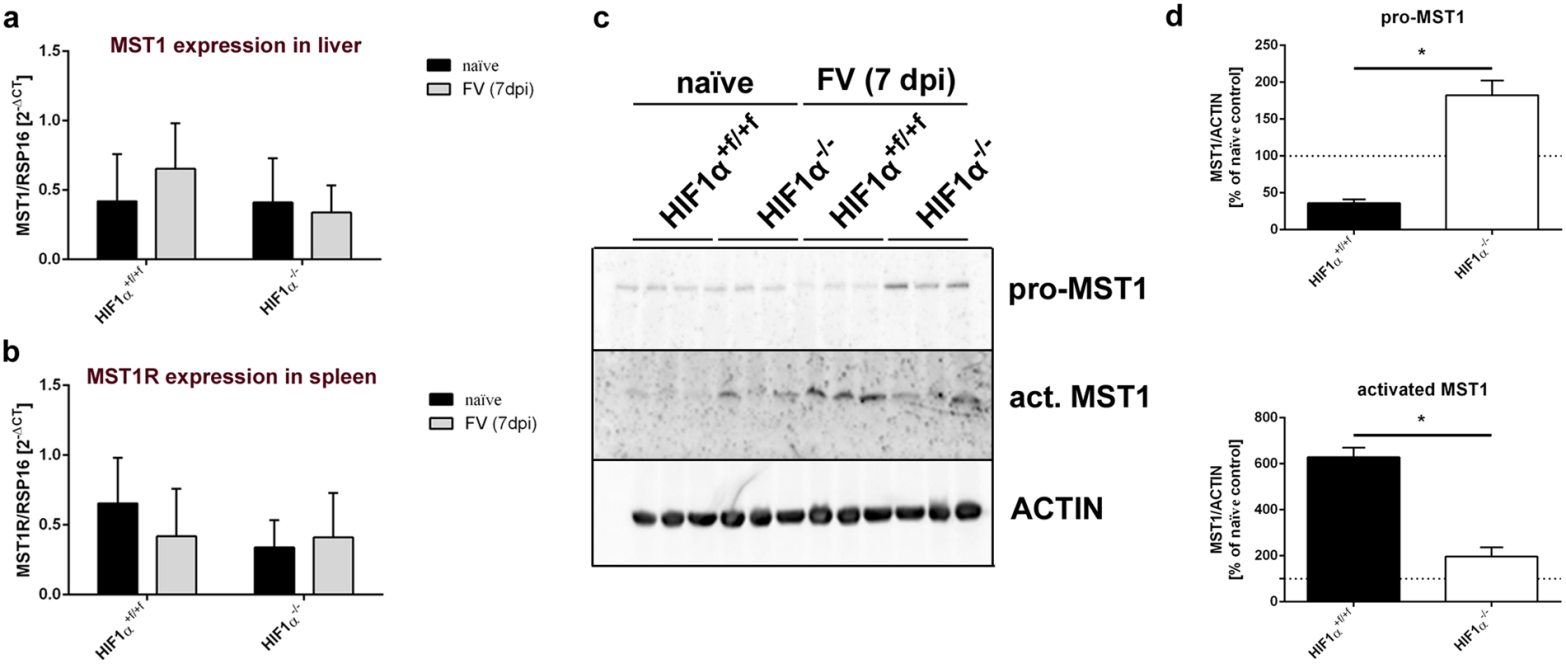
Friend virus infection leads to activation of macrophage-stimulating 1. Spleens and livers of naïve and Friend virus (FV)-infected wild-type (WT) and knockout (KO) mice were removed 7 days after infection (dpi). Next, mRNA was isolated, and real-time polymerase chain reaction (PCR) was used to determine the expression of *macrophage-stimulating 1 (MST1)* in the liver (**a**) and *MST1 receptor (MST1R)* expression in the spleen (**b**). Proteins were isolated from spleens, and the pro-form and activated isoforms of MST1 were quantified by Western blot analysis (**d**). Expression of MST1 was normalised to ACTIN and is depicted in comparison to naïve control animals of the appropriate genotype (**d**). Data were analysed with Student’s t-test (mean + SE). n = 3. *P = 0.05.

Pro-MST1 is produced in the liver and can be activated by the membrane-type serine protease 1 (MT-SP1) that is expressed on macrophages^36^. We determined whether MST1 is activated in the spleen after FV infection. Protein and mRNA from spleen and liver were isolated at 7 dpi, and real-time polymerase chain reaction (RT-PCR) and Western blot analyses were performed. RT-PCR analyses showed that neither relative *MST1* mRNA expression in the liver nor *MST1R* mRNA expression in the spleen changed during FV infection (Fig. 4a and b). However, Western blot analyses found increased MST1 protein levels after FV infection. The uncleaved proform pro-MST1 was elevated in infected *HIF-1α* KO mice, whereas the activated form (beta-chain) was elevated only in WT mice (Fig. 4c and d). Moreover, a moderate increase in activated MST1 was also seen in naïve HIF-1α KO animals and was probably due to the moderate increase in macrophages in the spleens of these mice. With respect to virus infection, however, we conclude that the increased number of macrophages in spleens of WT mice leads to increased cleavage of pro-MST1 and, thus, to elevated levels of active MST1 during acute infection.

### Activated MST1 leads to increased erythroblast proliferation

Because MST1R plays a crucial role in FV susceptibility, we investigated the role of MST1 in erythroblast proliferation during FV infection. To do so, we developed an *in vitro* assay for FV infection. Spleen cells were isolated and embedded in droplets of Matrigel as a scaffold for more complex tissue growth. After 4 days, these Matrigel droplets, which we called spleenoids, were populated by spleen cells and were then transferred to a spinning bioreactor for enhancement of nutrient absorption. After 7 days in culture, the number of erythroblast cell populations in splenoids was very similar to the number of erythroblast cell populations in the spleen (Supplementary Fig. 2a).

**Figure 5:**
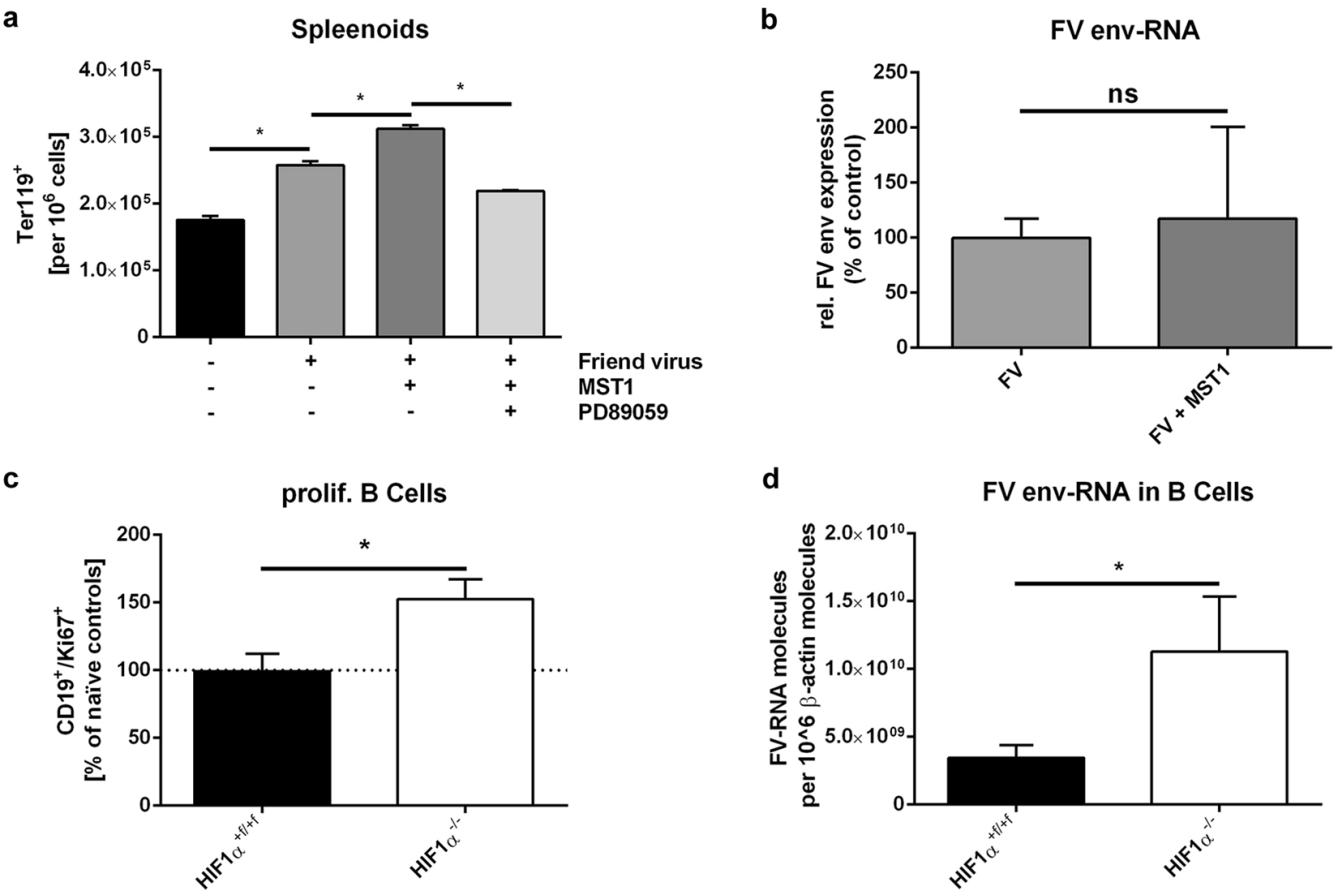
Activated macrophage-stimulating 1 leads to increased erythroblast proliferation. Splenic cells were isolated from naïve wild-type (WT) mice and cultivated as spleenoids. Spleenoids were treated with Friend virus (FV) either alone or in combination with 10 ng/mL macrophage-stimulating 1 (MST1) and 1 µM mitogen-activated protein kinase (MEK) inhibitor PD89059. Four days after infection, the number of erythrocytes (Ter119^+^) was determined by flow cytometry (**a**). Viral load was determined by real-time polymerase chain reaction (PCR) analysis. Expression of *FV envelope (env)* RNA was evaluated and normalized to β-actin expression (**b**). To determine whether B cells proliferate after FV infection in WT and knockout (KO) mice, we performed double staining for CD19 and the proliferation marker Ki67 and analysed proliferating B cells by flow cytometry. The number of Ki67^+^ CD19^+^ B cells is shown relative to the number in naïve control animals (**c**). Viral load in isolated B cells (7 days after infection [dpi]) was determined by real-time PCR analysis. Expression of *FV env*-RNA was evaluated and normalized to β-actin expression (**d**). Data were analysed with Student’s t-test (mean + SE). n = 3–10. *P = 0.05; ns, not significant.

For FV infection, spleenoids were infected *in vitro* before being transferred to the spinning bioreactor. Three days after FV infection, we determined the percentage of Ter119^+^ erythroblasts by using flow cytometry. FV infection resulted in higher numbers of erythroblasts in WT and *HIF-1α* KO spleenoids than in naïve control spleens (Supplementary Fig. 2b). Infection with FV and additional stimulation with recombinant activated MST1 led to a further increase in the number of erythroblasts. This increase was blocked by administration of the MEK inhibitor PD89059, a finding suggesting that this increase is caused by MST1R activation (Fig. 5a). Notably, RT-PCR analyses showed that MST1 did not increase the expression of *FV envelope* RNA in spleenoids at 3 dpi (Fig. 5b).

Because erythroblast proliferation after FV infection was prevented in *HIF-1α* KO mice by a reduction in the amount of activated MST1, we examined the FV-infected cell populations *in vivo*. B cells are another important target cell population, in addition to erythroblasts^37^. Because the decrease in the number of B cells in WT mice after FV infection was not seen in KO animals (see Fig. 2f), we examined the proliferation of B cells after infection (at 7 dpi) with the proliferation marker Ki67. The frequencies of proliferating B cells in WT mice did not change during infection. In contrast, the numbers of proliferating B cells increased by almost 50% in KO mice (Fig. 5c). Moreover, the expression of *FV envelope* RNA in isolated B cells was significantly higher in *HIF-1α* KO mice than in their WT littermates (Fig. 5d).

Taken together, these findings show that MST1 increases the proliferation of erythrocyte precursor cells during FV infection.

## Discussion

In this study, we investigated whether macrophage HIF-1α influences the outcome of acute Friend retrovirus infection. The role of macrophages in FV infection is largely unknown. Early studies of FV infection showed that macrophages play an integral role in both the pathogenesis and the suppression of FV disease. It has been shown that phagocytic and migratory functions of peritoneal macrophages from FV-infected mice are depressed^38^. A study by Marcelletti and colleagues found that the transfer of normal resident peritoneal macrophages to leukemic progressor mice causes regression of FV infection^39^. Additionally, macrophages have been shown to directly influence the growth and development of mature erythroid progenitors in normal and erythroleukaemic mice^40–42^. However, this mechanism is not involved in the EPO-independent erythroblast proliferation in FV leukemic mice^43,44^.

In the study reported here, we found a novel mechanism of FV-induced EPC proliferation. FV infection led to increased spleen weight in *HIF-1α* ^+*f/*+*f*^ (WT) and *HIF-1α* ^+*f/*+*f*^ × *LysM-Cre* (KO) mice at 7 and 10 dpi, and viral load was highest at 7 dpi. This course of FV infection is in line with that found by previous studies^21,33,45^. However, there were no differences in the progression of FV infection between WT and *HIF-1α* KO mice (Fig. 1).

Although the role of HIF-1α in macrophages during inflammatory and antibacterial activities has been addressed in several studies^13–15^, little is known about HIF-1α and viral infection. Pimonidazole staining showed that acute FV infection leads to an increase in hypoxic areas in the spleen (Fig. 3). A previous study found that infections with the parasite *Schistosoma mansoni* can lead to hypoxic areas and HIF-1α stabilization in murine spleens^46^. Our results showed that HIF-1α is stabilised in the spleen of FV-infected animals but is rapidly degraded after reoxygenation (see Fig. 3). Moreover, *HIF-1α* mRNA expression is not induced during infection.

These findings led us to conclude that HIF-1α is stabilised because of the hypoxic tissue environment that follows FV infection, not directly by the virus. This conclusion contrasts with those associated with other viral infections. Wakisaka and colleagues showed that the latent membrane protein 1 (LMP-1) of EBV leads to stabilisation of HIF-1α protein during chronic infections under normoxic conditions^47^. Moreover, the hepatitis B virus x (HBx) protein enhances the activity of HIF-1α under normoxic conditions^48^.

Because of hypoxic tissue, macrophages must move against oxygen gradients to infiltrate the spleen after FV infection. We found that *HIF-1α* KO macrophages exhibited impaired invasion capabilities under hypoxic conditions (Fig. 3). This finding mirrors the situation *in vivo*: fewer macrophages were detected in the spleens of *HIF-1α* KO mice than in the spleens of WT mice. It is also in line with findings reported by Cramer *et al*., who showed that the loss of HIF-1α in peritoneal macrophages leads to impaired migration and invasion capabilities *in vitro* ^13^.

Additionally, we found a significant decrease in the number of erythroblasts in *HIF-1α* KO mice compared to their WT littermates during FV infection (Fig. 1). To elucidate the underlying mechanism, we looked for MST1, a factor known to be implicated in EPC proliferation^32^. The *FV2* locus, which determines whether SFFV-infected erythroblasts proliferate in response to the retroviral envelope protein gp55, encodes *MST1R*. We found that MST1 exerts a profound effect on FV-mediated EPC proliferation. MST1 is constitutively transcribed in hepatocytes and circulates in the blood in an uncleaved proform. It is activated locally by cleavage into an alpha chain and a beta chain directly on the surface of macrophages. We found that the amount of MST1 protein in the spleen is elevated 7 days after FV infection (Fig. 4).

Compared to WT mice, naïve HIF-1α KO mice exhibit higher levels of activated MST1 but lower levels of pro-MST1. We hypothesize that this finding is due to a slight but not statistically significant increase in the number of macrophages in naïve control mice. However, we found an increase in activated MST1 after FV infection in WT mice only. In contrast, the reduced numbers of infiltrated macrophages resulted in higher levels of pro-MST1 in *HIF-1α* KO mice than in WT controls (Fig. 4). We observed enhanced EPC proliferation in spleenoids *in vitro* during an ongoing FV infection after the addition of active MST1. In addition, this increase appeared to be mediated by induction of the mitogen-activated protein (MAP) kinase pathway (Fig. 5). Furthermore, activation of this pathway did not result in increased amounts of FV RNA in infected spleenoids, a finding mimicking the results *in vivo*, in which reduced amounts of activated MST1 in *HIF-1α* KO mice did not decrease viral load (Fig. 1b). This finding appears to be due to an increase in FV-induced B-cell proliferation in KO animals (Fig. 5). It also explains why the reduced numbers of erythroblasts and macrophages in KO animals did not result in decreased spleen weights (Fig. 1).

It has already been suggested that MST1-activated receptor may cause EPOR tyrosine phosphorylation and that MST1R/EPOR cross-talk may be crucial for regulation of normal erythropoiesis and the development of erythroleukaemia^49^. Moreover, it has been previously shown that direct activation of MST1R is sufficient to substitute EPO in EPC expansion^50^ and that MST1 can enhance the proliferation of EPCs in response to EPO^32^. However, ours is the first study to show that MST1 plays a role in FV-induced erythroblast proliferation.

In summary, we found that FV infection leads to increased spleen weights in *HIF-1α* ^+*f/*+*f*^ (WT) and *HIF-1α* ^+*f/*+*f*^ × *LysM-Cre* (KO) mice after 7 days, but the infection leads to massive erythroblast proliferation in WT mice only. Moreover, only WT mice exhibit a strong increase in the number of macrophages after infection. This increase is caused by impaired invasion capabilities of *HIF-1α* KO macrophages under hypoxic conditions during retroviral infection. The absence of macrophages from KO mice results in decreased levels of activated MST1, which is an important factor in FV-induced EPC proliferation.

## Methods

### Mice

Inbred C57BL/6J mice with loxP sites flanking exon 2 of the *HIF-1α* gene (*HIF-1α* ^+*f/*+*f*^, purchased from The Jackson Laboratory, Bar Harbor, ME, USA) were crossbred with mice with a lysozyme 2 gene (*Lyz2*) promoter (*LysM*)-driven Cre recombinase (*HIF-1α* ^+*f/*+*f*^ × *LysM-Cre*); this crossbreeding achieved a myeloid-specific *HIF-1α* KO. Exon 2 encodes for the DNA binding site of translated HIF-1α protein. Littermates negative for Cre recombinase (*HIF-1α* ^+*f/*+*f*^) served as control animals. All mice were maintained under pathogen-free conditions. Transgenic mice were backcrossed on a C57BL/6 background and were resistant to FV-induced leukaemia. All mice were 8 to 16 weeks of age at the beginning of the experiments.

Animal experiments were performed in full accordance with the German law for animal welfare and with institutional regulations for animal breeding and handling and were approved by the State Agency for Nature, Environment and Consumer Protection North Rhine-Westfalia (file reference, 84-02.04.2013.A317).

### Virus and viral infection

The FV stock used in these experiments was FV complex containing B-tropic Friend murine leukaemia helper virus and polycythaemia-inducing SFFV^51^. The stock was prepared as a 10% spleen cell homogenate from BALB/c mice infected 14 days previously with 3,000 spleen focus-forming units (SFFU) of non-cloned virus stock. Experimental mice were injected intravenously with 0.2 mL phosphate-buffered saline (PBS) containing 20,000 SFFU of FV. The virus stock was free of lactate dehydrogenase–elevating virus.

### Infectious centre assay

Infectious centre assays were performed as previously described^33^.

### Cell-surface and intracellular staining by flow cytometry

Spleen cells were stained with fluorochrome-conjugated antibodies and analysed with a FACSCalibur flow cytometer and FACSDiva software (BD Biosciences, Oxford, UK). Cells were stained with the following antibodies: anti-Ter119-PE-Cy7 (TER-119; eBioscience, Inc., San Diego, CA, USA), anti-F4/80–fluorescein isothiocyanate (FITC; CI:A3-1), anti-CD11b-PE-Cy7 (M1/70), anti-CD11c-APC (N418), anti-CD4-APC (GK1.5), anti-CD8-PB (53-6.7), anti-CD19-FITC (6D5) (all from BioLegend, San Diego, CA, USA), and anti-Ki67-SAV (SoIA15; eBioscience). Dead cells were excluded with fixable viability dye (FVD)-eFluor 780 (eBioscience).

### Bone marrow–derived macrophages

BMDMs were isolated from the femurs and tibias of mice as previously described^52^. Briefly, bone marrow was flushed from the bone cavity with a 23 G needle and syringe (BD Biosciences) containing macrophage medium consisting of minimal essential medium (MEM) plus 100 U/mL penicillin/streptomycin, 5 mM L-glutamine, 1% (v/v) sodium pyruvate (all from Invitrogen, Waltham, MA, USA), 1 mM HEPES (Sigma-Aldrich, Munich, Germany), and 0.6 mM tissue culture grade β-mercaptoethanol, supplemented with 10% (v/v) fetal calf serum (FCS; Biochrom GmbH, Berlin, Germany). Cells in the bone marrow flush were plated onto non-treated cell culture flasks (BD Bioscience) in macrophage medium containing 10% (v/v) L929 cell-conditioned medium. After 24 hours of incubation at 37 °C and 5% CO_2,_ the non-adherent population was replated into a 6-well culture plate (Sarstedt, Nuembrecht, Germany) at a density of 1 × 10^6^ cells per well with fresh macrophage medium. The adherent population was discarded. After an additional five days in culture, the non-adherent population was discarded, and remaining adherent BMDMs were harvested for experiments.

### Viability of macrophages under hypoxic conditions

Isolated WT and *HIF-1α* KO BMDMs were incubated for 72 hours under normoxic (20.9% O_2_) or hypoxic (1% O_2_) conditions. Briefly, dead macrophages were stained with 0.2 µg/mL DAPI (AppliChem GmbH, Darmstadt, Germany) for 2 minutes with the DAPI exclusion test^53^. Subsequently, cells were examined with a fluorescence microscope (Axiovert 200 M, Carl Zeiss Microscopy GmbH, Jena, Germany), and stained cells were counted manually in relation to the total number of cells in the field.

### Inverted macrophage invasion assay

The 3D inverted invasion assay was performed as previously described^35^. Briefly, 100 mL of Matrigel (BD Biosciences), mixed 1:1 with ice-cold PBSand supplemented with 50 mg/mL bovine plasma fibronectin (Invitrogen), was transferred to a transwellinsert (8-mm pore; Corning Incorporated, Corning, NY, USA) to polymerise at 37 °C. After polymerisation, transwell inserts were inverted, and 5 × 10^4^ WT or *HIF-1α* KO BMDMs were applied directly to the lower side of the filter insert and allowed to adhere for 2 hours. Finally, inserts were placed into a chamber containing macrophage medium and incubated for 72 hours at 37 °C and 5% CO_2_ under either normoxic (20.9% O_2_) or hypoxic (1% O_2_) conditions for invasion into the gel. Cells were then stained with 4 mM calcein-AM (Invitrogen). Serial confocal optical sections of the Matrigel/fibronectin gel were captured with a Zeiss LSM510 laser scanning confocal microscope (Zeiss). For each experimental condition, the inverted invasion assay was performed in duplicate, and confocal data were collected from 3 fields of view per transwell (total, 6 fields of view). Experiments were repeated with cells independently isolated from 3 mice per genotype. Invasion data were quantified with ImageJ software (National Institutes of Health, Bethesda, MD, USA).

### Immunostaining

Sections (5 µm) of spleen tissue were stained with hematoxylin (Merck, Darmstadt, Germany) and eosin (Sigma-Aldrich).

### Pimonidazole staining

For detection of hypoxia, pimonidazole was injected intraperitoneally (i.p.). One hour later, spleens were removed, fixed in 4% paraformaldehyde (Sigma-Aldrich), and embedded in paraffin. Sections (5 μm) were cut from the paraffin blocks, deparaffinised with xylene, and rehydrated in a graded series of alcohols. Immunohistochemistry was performed as previously described^46^.

### Protein isolation and Western blot analyses

So that rapid degradation of HIF-1α protein could be avoided, spleens were removed from the animals, and proteins were isolated in a hypoxic workstation (1% O_2_). To determine whether HIF-1α was stabilized because of a hypoxic environment within the spleen after FV infection, a portion of the spleen was removed from the workstation and incubated with 20.9% O_2_ for 10 minutes for induction of reoxygenation and, thus, HIF-1α degradation. Western blot analysis was performed as previously described^54^, and protein was loaded (up to 100 µg per lane). The following polyclonal antibodies were used: anti-HIF-1α (Cayman Chemical, Ann Arbor, MI, USA), anti-MST1 (pro-MST1; Abcam, Cambridge, UK), anti-MST1 (beta-chain, Abcam), and anti-ACTIN (Sigma-Aldrich).

### RNA isolation and real-time polymerase chain reaction analyses

Total RNA was isolated from spleens and livers with the RNeasy Mini Kit (Qiagen, Hilden, Germany). RT-PCR was performed as previously described^54^ with SYBR green fluorescent dye (Eurogentec, Verviers, Belgium) and the iQ5 Real-time PCR Detection System (Bio-Rad Laboratories GmbH, Munich, Germany). Amounts of complementary DNA were normalised to ribosomal protein, and expression was calculated with the 2^−ΔΔCT^ method^55^. The following primers were used: HIF-1α sense, GAA ATG GCC CAG TGA GAA AA; HIF-1α antisense, CTT CCA CGT TGC TGA CTT GA; MST1 sense, TGC TAT ACC TTG GAC CCG GA; MST1 antisense, TCA AAC ACC ACC TGG TCT GG; MST1R sense, AGC ATG GCA CTT CAC GCT AT; MST1R antisense, GTT GTT CCA CAA ACT GCC CC; RSP16 sense, AGA TGA TCG AGC CGC GC; RSP16 antisense, GCT ACC AGG GCC TTT GAG ATG GA. FV envelope RNA loads were measured as previously described and were normalized to β-actin expression levels^56^.

### B-cell isolation

Seven days after FV infection, splenic B cells were isolated with CD19 microbeads (Miltenyi Biotec, Bergisch Gladbach, Germany) according to the manufacturer’s instruction.

### Spleenoid culture

Single-cell suspensions were generated from spleens by filtering cells through a 70-µm nylon mesh (BD Biosciences, San Jose, CA, USA) and diluted with PBS to a concentration of 1 × 10^8^ cells per mL. For each spleenoid, 10 µL of the cell suspension was transferred into a 1.5-mL reaction tube and centrifuged at 1500 relative centrifugal force (rcf) for 5 minutes. Supernatant was discarded, and cells were resuspended in ice-cold Matrigel (BD Biosciences).

For each spleenoid, 15 µL of the cell/Matrigel suspension was transferred by pipetting onto a sheet of Parafilm (American National Cam, IL, USA) with small 3-mm dimples. The droplets were allowed to gel at 37 °C and were subsequently removed from the Parafilm and grown in spleenoid medium containing Aim-V medium (Invitrogen) supplemented with 10% FCS, 4 mM L-glutamine, 0.6 mM tissue culture grade β-mercaptoethanol, and 100 U/mL penicillin/streptomycin. After 3 days of stationary growth, the tissue droplets were transferred to a spinning bioreactor and cultivated for 4 additional days. For FV infections, spleenoids were infected with 2,000 SFFU injected directly into the organoid; additionally, 40,000 SFFU were added to the culture medium (50 mL). For co-stimulation, infected spleenoids were treated with 10 ng/mL MST1 (R&D Systems, Minneapolis, MN, USA). For MEK inhibition, spleenoids were cultivated as described above in the presence of 10 ng/mL MST1 and 1 µM MEK inhibitor (PD98059, Cell Signaling Technology, Inc., Danvers, MA, USA).

### Statistical analyses

Data were analysed with ANOVA and Tukey’s multiple comparison test or with Student’s t-test (GraphPad Prism software; GraphPad Software, Inc., La Jolla, CA, USA).

## Electronic supplementary material

Supplementary Information
