# Supplementary material for: Hypoxia-inducible factor 1α is Essential for Macrophage-mediated Erythroblast Proliferation in Acute Friend Retrovirus Infection
Source: Sci Rep. 2017 Dec 8;7:17236. doi: 10.1038/s41598-017-17324-y (PMC5722883; doi:10.1038/s41598-017-17324-y)
Supplement: Supplementary file 1 — Supplementary Information [file 41598_2017_17324_MOESM1_ESM.doc]

**Hypoxia-inducible factor-1α is Essential for Macrophage-mediated Erythroblast Proliferation in Acute Friend Retrovirus Infection**

**Timm Schreiber^1^, Theresa Quinting^1^, Ulf Dittmer^2^, Joachim Fandrey^1*§^, and Kathrin Sutter^2§^**

^1^University of Duisburg-Essen, Institute of Physiology, Essen, Germany.

^2^University of Duisburg-Essen, Institute of Virology, Essen, Germany.

*corresponding author: Joachim.fandrey@uni-due.de

^§^contributed equally

**Supplemental Figures**

**
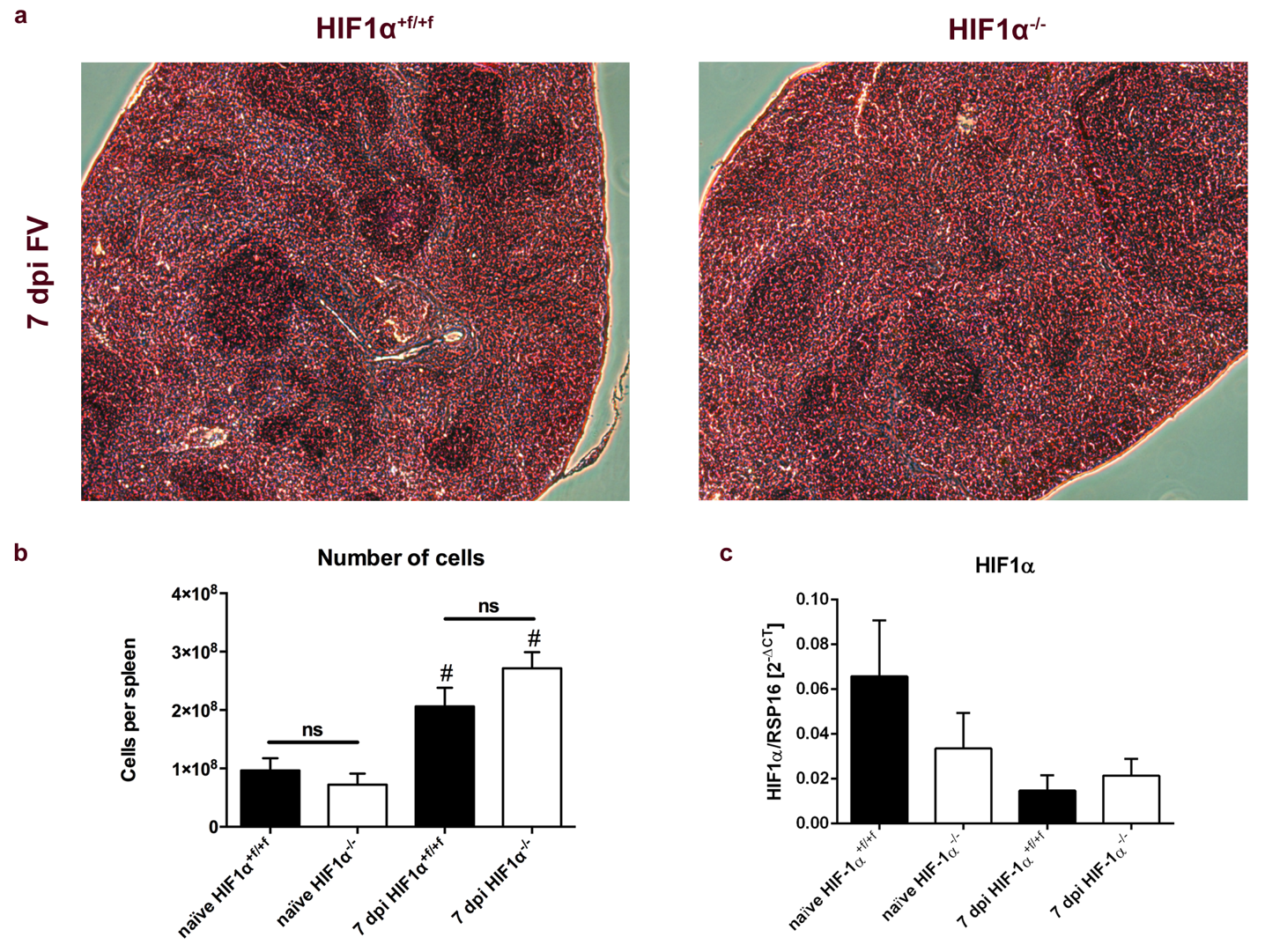
**

**Supplementary Figure 1: Acute Friend virus infection did not result in structural alterations in splenic architecture in wild-type or knockout mice.** Spleens of wild-type (WT) and knockout (KO) mice were removed 7 days after Friend virus (FV) infection. Sections of spleen tissue were stained with haematoxylin and eosin for morphological analyses (**a**). Cell numbers after FV infection were determined by isolating cells and counting them manually with a Neubauer chamber (**b**). For expression analyses, mRNA was isolated, and real-time polymerase chain reaction for HIF1α was performed (**c**). Data were analysed with Student’s t-test (mean ± SEM). n = 3. #, P = 0.05 compared to naïve mice; *, P = 0.05 for comparison between WT and KO mice; ns, not statistically significant.

**
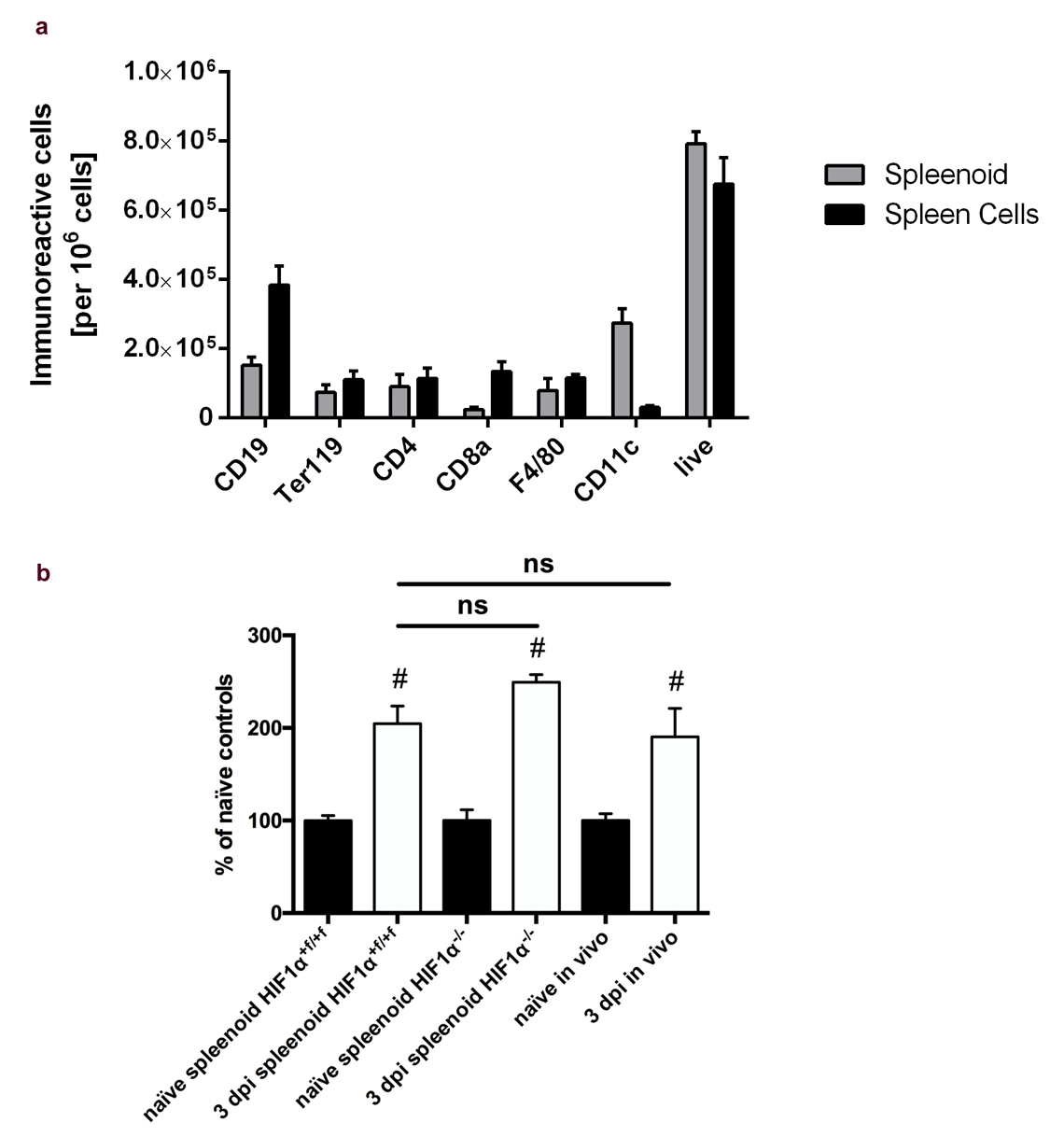
**

**Supplementary Figure 2: The cellular composition of spleenoids mimics the composition *in vivo*.** Splenic cells were isolated and cultivated as spleenoids. After 3 days in culture, spleenoids were transferred to a spinning bioreactor. After 7 days in culture, cells were isolated and subpopulations of cells were analysed by flow cytometry. The following antibodies were used for cell population analysis: CD19, B cells; Ter119, erythrocyte precursor cells; CD4, helper T (T_h_) cells; CD8a, cytotoxic T lymphocytes (CTLs); F4/80, macrophages; CD11c, dendritic cells (**a**, mean ± SEM). To exclude a direct effect of myeloid hypoxia-inducible factor 1α (HIF1α) on erythroblast proliferation, we infected spleenoids from knockout mice and analyzed Ter119^+^ cells by flow cytometry (**b**). For comparison, we included *in vivo* data from wild-type (WT) mice at 3 days after infection (dpi). Data were analysed with Student’s t-test (mean ± SEM). n = 3-6. #, P = 0.05 compared to naïve mice; *, P = 0.05 for comparison between WT and knockout (KO) mice; ns, not statistically significant.


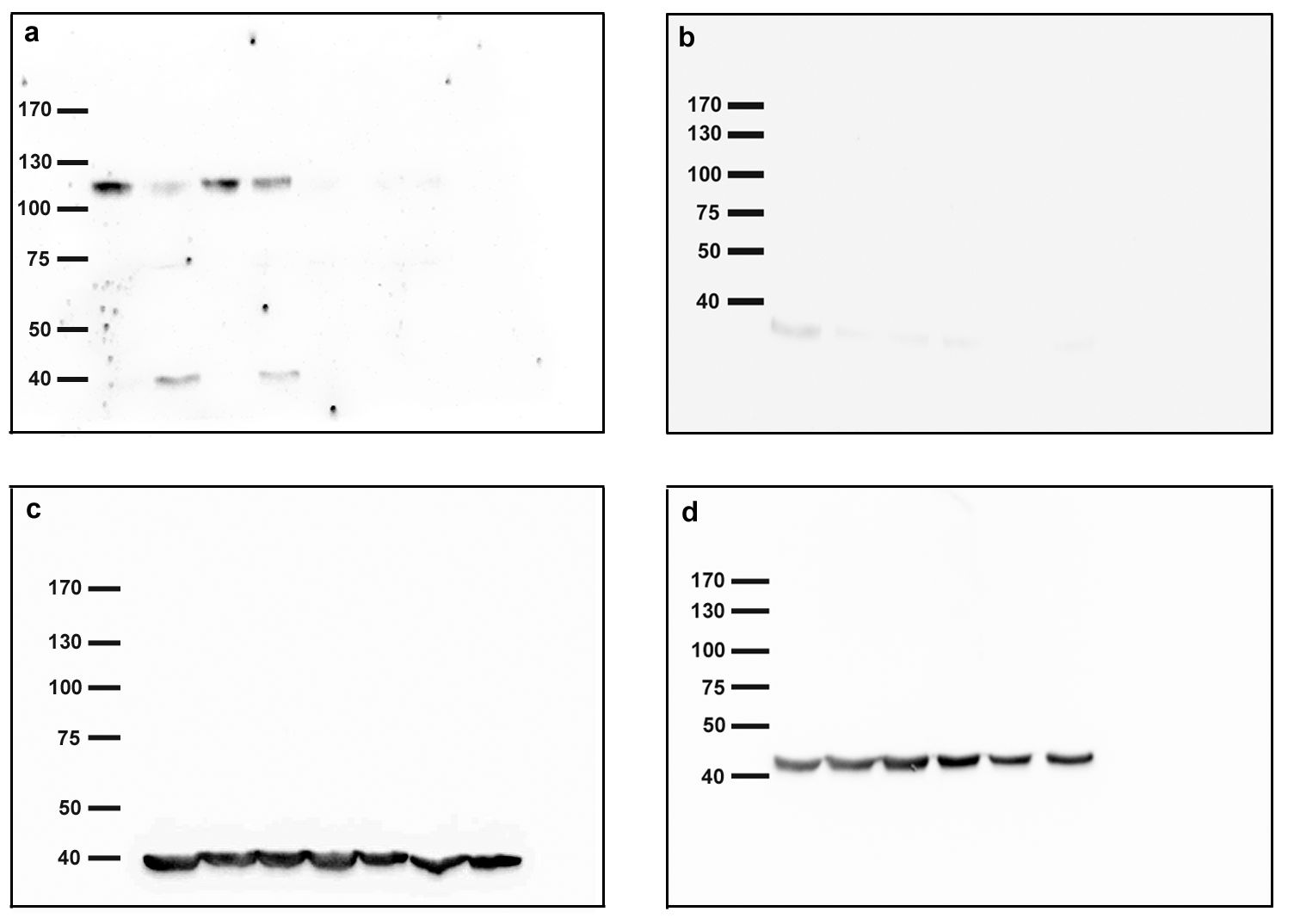


**Supplementary Figure 3: Original full size Western blots from Figure 3.** HIF-1α, spleen in vivo (**a**). HIF-1α, reoxygenated ex vivo (**b**). Actin, spleen in vivo (**c**). Actin, reoxygenated ex vivo (**d**).


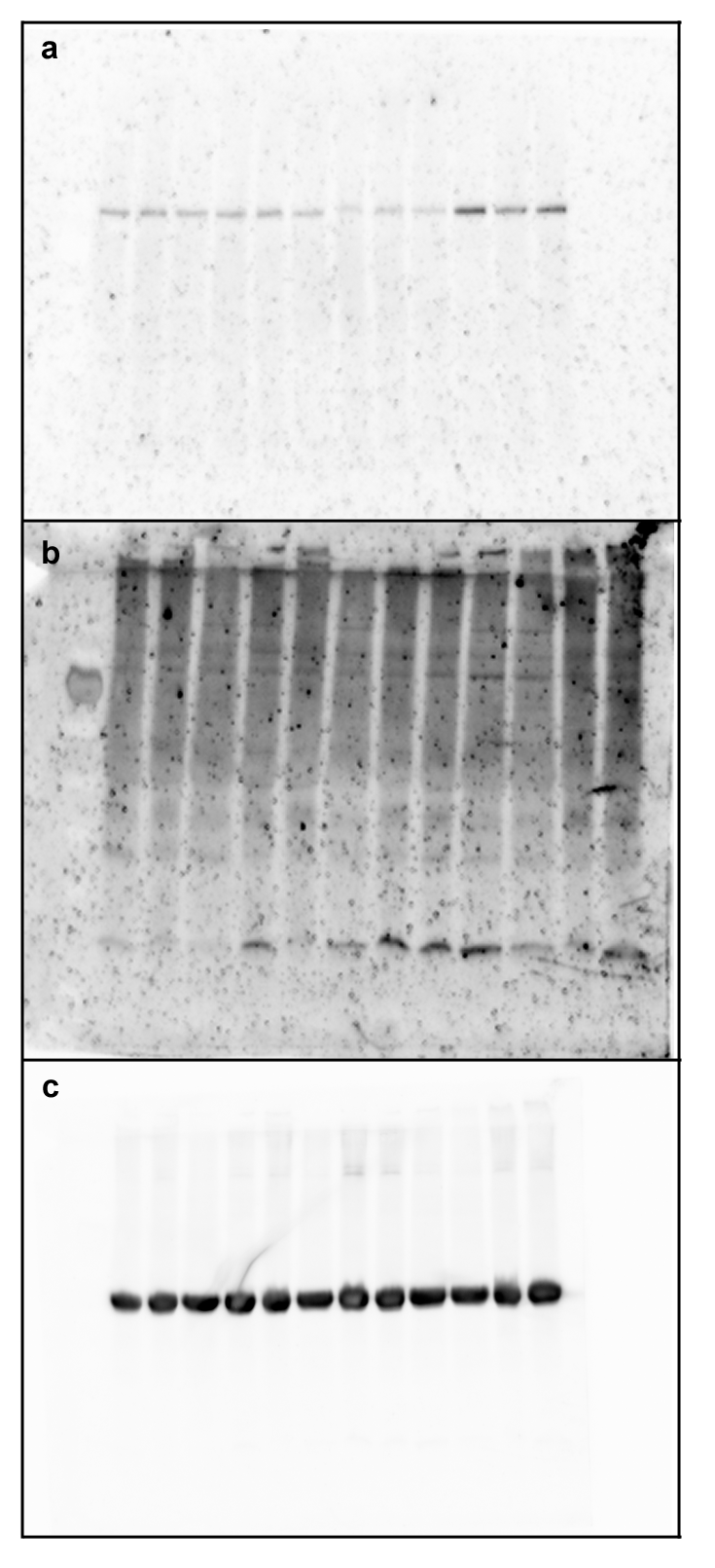


**Supplementary Figure 4: Original full size Western blots from Figure 4.** Pro-MST1 (**a**). act. MST1 (**b**). Actin (**c**).
